# Correction: New proteomic biomarkers identified in plasma extracellular vesicles in sarcoidosis: a case-control matched study

**DOI:** 10.3389/fimmu.2026.1931341

**Published:** 2026-07-20

**Authors:** Runzhen Zhao, Nan Miles Xi, Gabrielle Lea, Emily R. Gilbert, Kamala Vanarsa, Mark Qiao, Dee Zhang, Jiwang Zhang, Chandra Mohan, Marc A. Judson, Laura L. Koth, Hong-Long Ji

**Affiliations:** 1 Department of Surgery, Stritch School of Medicine, Loyola University Chicago Health Sciences Division, Maywood, IL, United States; 2 Burn and Shock Trauma Research Institute, Stritch School of Medicine, Loyola University Chicago Health Sciences Division, Maywood, IL, United States; 3 Department of Mathematics and Statistics, Loyola University Chicago, Chicago, IL, United States; 4 Biomedical Engineering & Medicine, University of Houston, Houston, TX, United States; 5 Department of Medicine, Loyola University Chicago, Maywood, IL, United States; 6 Department of Cancer Biology, Loyola University Chicago, Maywood, IL, United States; 7 Department of Medicine, Albany Einstein College of Medicine, Albany, NY, United States; 8 Department of Medicine, University of California, San Francisco, San Francisco, CA, United States; 9 Department of Microbiology and Immunology, Stritch School of Medicine, Loyola University Chicago Health Sciences Division, Maywood, IL, United States

**Keywords:** biomarker, extracellular vesicles, machine learning, proteome wide, sarcoidosis

Author “Gabrielle Lea” was erroneously spelled as “Lea Gabby.”

The author contributions statement was erroneously given as:

“RZ: Data curation, Formal analysis, Funding acquisition, investigation, Methodology, Project administration, Resources, Writing – original draft, Writing – review & editing. NX: Data curation, Formal analysis, Investigation, Methodology, Validation, Visualization, Writing – original draft. LG: Data curation, Formal analysis, Writing – original draft. EG: Resources, Writing – review &editing. KV: Data curation, Formal analysis, Writing – review & editing. MQ: Data curation, Formal analysis, Writing – original draft. DZ: Resources, Validation, Writing – review & editing. JZ: Writing – review & editing. CM: Writing – review & editing. MJ: Writing – review & editing. LK: Funding acquisition, Writing – review & editing. H-LJ: Conceptualization, Funding acquisition, Investigation, Methodology, Project administration, Resources, Supervision, Writing – original draft, Writing – review & editing.”

The correct **Author Contributions** statement is:

“RZ: Data curation, Formal analysis, Funding acquisition, investigation, Methodology, Project administration, Resources, Writing – original draft, Writing – review & editing. NX: Data curation, Formal analysis, Investigation, Methodology, Validation, Visualization, Writing – original draft. GL: Data curation, Formal analysis, Writing – original draft. EG: Resources, Writing – review &editing. KV: Data curation, Formal analysis, Writing – review & editing. MQ: Data curation, Formal analysis, Writing – original draft. DZ: Resources, Validation, Writing – review & editing. JZ: Writing – review & editing. CM: Writing – review & editing. MJ: Writing – review & editing. LK: Funding acquisition, Writing – review & editing. H-LJ: Conceptualization, Funding acquisition, Investigation, Methodology, Project administration, Resources, Supervision, Writing – original draft, Writing – review & editing.”

The original version of this article has been updated.

